# Effectiveness of the Novel Herbal Medicine, KIOM-MA, and Its Bioconversion Product, KIOM-MA128, on the Treatment of Atopic Dermatitis

**DOI:** 10.1155/2012/762918

**Published:** 2012-02-01

**Authors:** Tae Ho Chung, Tae Jin Kang, Won-Kyung Cho, Ga Young Im, Geum Seon Lee, Min Cheol Yang, Chang-Won Cho, Jin Yeul Ma

**Affiliations:** ^1^Center for Herbal Medicine Improvement Research, Korea Institute of Oriental Medicine (KIOM), 483 Expo-ro, Yuseong-gu, Daejeon 305-811, Republic of Korea; ^2^College of Pharmacy, Sahmyook University, 26-21 Kongnung 2-dong, Nowon-gu, Seoul 139-742, Republic of Korea; ^3^Regional Food Industry Research Group, Korea Food Research Institute, Sungnam 463-746, Republic of Korea

## Abstract

This study was conducted to determine if oral administration of the novel herbal medicine, KIOM-MA, and its *Lactobacillus acidophilus*-fermented product, KIOM-MA128, has therapeutic properties for the treatment of atopic dermatitis (AD). Using AD-induced BALB/c mice by Ovalbumin and aluminum hydroxide, the effectiveness of KIOM-MA and KIOM-MA128 on AD was evaluated. Oral administration of KIOM-MA and KIOM-MA128 reduced major clinical signs of AD including erythema/darkening, edema/papulation, excoriations, lichenification/prurigo, and dryness. Interestingly, KIOM-MA128 more significantly improved AD-related symptoms including decrease of IgE level in the plasma as well as reduction of scratching behavior, skin severity in the AD BALB/c model. HPLC analysis showed the significant changes in the constituent patterns between KIOM-MA and KIOM-MA128. Our results suggest that both KIOM-MA and KIOM-MA128 have potential for therapeutic reagent for the treatment of AD, and further, the efficacy is significantly enhanced by *L. acidophilus* fermentation via increases in its indicator molecule.

## 1. Introduction

Atopic dermatitis (AD) is a chronic inflammatory skin disease associated with cutaneous hyperreactivity to environmental triggers that are innocuous to normal nonatopic individuals [1]. Various studies have shown that atopic dermatitis has a complex etiology, with activation of multiple immunological and inflammatory pathways [2]. In addition, several studies have suggested that atopic dermatitis is the cutaneous manifestation of a systemic disorder that also gives rise to asthma, food allergy, and allergic rhinitis [3, 4]. These conditions are all characterized by elevated serum IgE levels and peripheral eosinophilia [5].

AD has long been considered a systemic disease for which there are few satisfactory systemic therapies that do not involve glucocorticoids [6]. In general, topically applied medication of adequate potency, mainly corticosteroids, has been standard for the vast majority of patients with AD [6]. Although intermittent use of topical corticosteroids is highly effective, the repeated use of steroids is not desirable due to the systemic adverse effects their long-term use can induce [7, 8]. Accordingly, research has begun to identify steroid-free alternative therapeutic agents with better efficacy and safety [9].

There has been increased interest in the use of traditional herbal medicine to develop new therapeutic agents without a steroid for AD treatment [10]. In addition, recent double-blind, placebo-controlled, crossover studies show considerably effective benefits in managing clinical signs of AD [11–14]. However, it is important to investigate the active principles of the herbal medicines for quality control and to determine their real therapeutic value in modern pharmacology [15, 16]. Recent studies have suggested that fermentation of herbal extracts may affect the therapeutic potential due to their increased absorptive effect [17].

In this study, we report that newly formulated herbal medicine KIOM-MA possesses strong antiatopic activity and KIOM-MA128, fermented form by *L. acidophilus*, exerts more potent inhibitory effect on AD.

## 2. Materials and Methods

### 2.1. Preparation and Fermentation of Novel Herbal Medicine

To develop a novel herbal medicine formula for the treatment of AD, over 100 herbal medical formulas were tested by the Center for Herbal Medicine Improvement Research at the Korea Institute of Oriental Medicine. A novel herbal medicine with antiatopic dermatitis effects, KIOM-MA (Glycyrrhizae Radix, Polygoni Cuspidati Rhizoma, Sophorae Radix, Cnidii Rhizoma, Arctii Fructus, etc., Korean Patent Application no. 10-2010-0093901, PCT/KR2010/007523), was identified during this testing. Water extracts were then prepared by steeping finely crushed herbs containing the KIOM-MA medical formulation as described previously [18], with some modification. Briefly, 1840.0 g of the KIOM-MA formula were placed in 18.4 L of distilled water and boiled for about three hours at 115°C. The extract was then filtered through a testing sieve (Aperture 500 *μ*m and 150 *μ*m). All herbal plant materials were purchased from Yeongcheon Oriental Herbal Market, Gyeongbuk Province, Republic of Korea.

Fermentation was conducted using *Lactobacillus acidophilus* (KFRI 128, KCTC 2182) donated from the Korea Food Research Institute (KFRI). Briefly, KIOM-MA water extract was autoclaved for 5 min, after which the pH was neutralized using the addition of 1 M NaOH. *L. acidophyllus* was prepared by culturing in MRS broth at 37°C under anaerobic conditions. The initial concentration of bacteria ranged from 10^5^ to 10^7^ CFU/mL depending on the substrate. KIOM-MA containing *L. acidophyllus* was fermented at 37°C for 48 h using MRS medium (10.0 g/L Peptone, 10.0 g/L Beef extract, 5.0 g/L Yeast extract, 20.0 g/L Glucose, 1.0 mL/L Tween 80, 2.0 g/L K_2_HPO_4_, 5.0 g/L Sodium acetate, 2.0 g/L Triammonium citrate, 0.2 g/L MgSO_4_·7H_2_O, 0.2 g/L MnSO_4_·4H_2_O, pH 6.2–6.6). The KIOM-MA128 was then passed through a 60 *μ*m nylon net filter (Millipore, MA, USA), precipitated overnight, lyophilized (supernatant), and stored in desiccators at room temperature prior to use.

### 2.2. HPLC Analysis of KIOM-MA and KIOM-MA128

Active principles separation was conducted using a reverse-phase HPLC system consisting of an HPLC pump, as previously described [19]. Fermented samples were monitored at 254 nm and the area responses were integrated. The major active compounds, isoflavones, were identified based on the retention time and the PDA spectra of the pre- and postfermentation herbal extracts were compared. Standard molecules (liquiritin, nodakenin, icariin, and decursin) were purchased from the Korea Food & Drug Administration. Glycyrrhizin was purchased from Tokyo Chemical Industry Co. (Tokyo, Japan). Decursinol angelate was purchased from Natural Product Chemistry BioTech Inc. HPLC grade solutions, water, acetonitrile, and glacial acetic acid were purchased from J. T. Baker Co. (NJ, USA).

### 2.3. Chromatographic Conditions

The high-performance liquid chromatography (HPLC) data were obtained using an Elite Lachrom analytical HPLC PDA system that included an L-2130 binary HPLC pump, an L-2200 autosampler, a column oven (L-2350), and a diode array UV/VIS detector (L-2455). The output signal of the detector was recorded using the EZchrom Elite software for Hitachi. For separation of the samples, an OptimaPak C 18 column (4.6 mm × 250 mm, 5 *μ*m, RS tech, republic of Republic of Korea) was employed and the PDA UV wavelength was 254 nm. The mobile phase was water and acetonitrile with a gradient elution containing 2% glacial acetic acid at a flow rate of 1.0 mL/min. The column temperature was maintained at 40°C (Table 1) and the injection volume of the samples was 10 *μ*L.

### 2.4. Preparation of Standard Solutions and Samples

To prepare the analytical samples, KIOM-MA and KIOM-MA128 (fermented KIOM-MA) powder were accurately weighed and dissolved in 100% H_2_O at a concentration of 40 mg/mL. Prior to analysis, the sample was filtered through a 0.45 *μ*m filter.

### 2.5. Animals

3-week-old male BALB/c mice were obtained from Hanlim Laboratory Animals Co. (Hwasung, Kyunggi-Do, Korea) and maintained for two weeks before the start of the experiments. All animals were maintained on a standard light-dark cycle at ambient temperature (23 ± 2°C) and humidity (55 ± 10%) with free access to chow pellets and water. The experimental groups, which consisted of 5–7 animals per drug and dose, were selected by a randomized schedule. Animal treatment and maintenance were conducted in accordance with the Principle of Laboratory Animal Care (NIH publication no. 85-23 revised in 1985) and the Animal Care and Use Guidelines of Sahmyook University, Republic of Korea.

### 2.6. Atopic Dermatitis Models

5-week-old BALB/c mice were anesthetized with ether and the dorsal skin was then shaved with a clipper and a shaver one day before experiments. The exposed dorsal region was treated with Ovalbumin and aluminum hydroxide to induce AD. Mice were randomly assigned to one of seven groups at the start of the experiment (*n* = 5): Group A, normal control; Group B, AD control; Group C, KIOM-MA treatment (50 mg/kg); Group D, KIOM-MA treatment (100 mg/kg); Group E, KIOM-MA-128 treatment (50 mg/kg); Group F, KIOM-MA-128 treatment (100 mg/kg); and Group G, dexamethasone (1 mg/kg). Following the last administration of KIOM-MA or KIOM-MA128 on day 14, the mice were sacrificed and the plasma IgE levels were measured.

### 2.7. Clinical Skin Severity Score

The dorsal skin of each mouse was photographed before, during, and after KIOM-MA or KIOM-MA128 treatment. The severity of AD-like dorsal skin lesions was assessed once a day as follows: dorsal lesions were evaluated for five symptoms, erythema/darkening, edema/papulation, excoriations, lichenification/prurigo, and dryness. Each symptom was graded from 0 to 3 (none, 0; mild, 1; moderate, 2; severe, 3). The clinical skin score was defined as the sum of the individual scores, ranging from 0 to 15.

### 2.8. Established Lesions and Scratching Behavior

The scratching behavior was recorded on video once a day for 11 consecutive days. Specifically, the number of times a mouse scratched the dorsal skin lesion within a period of 15 min was counted. Because the average number of scratches in each mouse varied daily, the scratching behavior was estimated as the percentage of the control calculated from the mean value of the no-treatment group.

### 2.9. Detection of Serologic IgE Concentration

Mice were sacrificed on 1, 3, 7, and 14 days after treatment of KIOM-MA or KIOM-MA128, and cardiac blood was collected from mice by thoracotomy into EDTA-treated tubes, after which plasma was separated by centrifugation at 3,000 g for 10 min at 4°C and stored at −80°C. The plasma levels of IgE were measured with ELISA kits (R & D System, Boston, MA, USA) according to the manufacturer's instruction.

### 2.10. Statistical Analysis

Experimental values are given as the means ± SEM. The statistical difference was determined by a two-sided Mann-Whitney *U*-test. A *P* < 0.05 was considered to indicate statistical significance.

## 3. Results

### 3.1. HPLC Analysis

**Figure 1:**
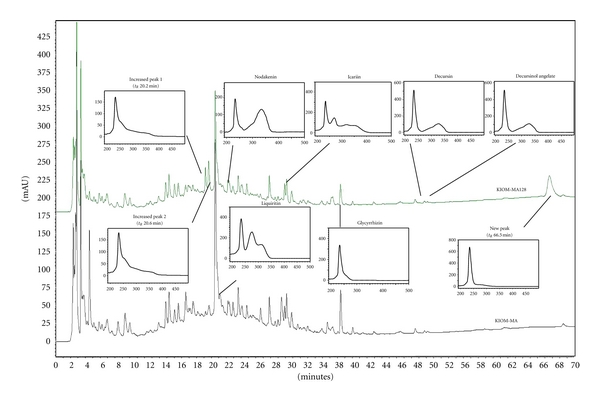
The HPLC chromatogram of KIOM-MA and KIOM-MA128 at 254 nm.

**Figure 2:**
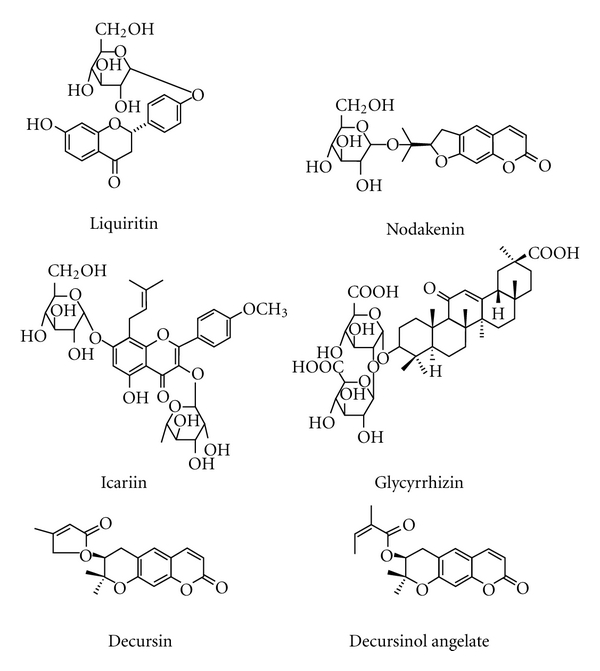
The chemical structure of six markers in KIOM-MA and KIOM-MA128.

Six maker compounds of KIOM-MA and KIOM-MA128 liquiritin (tR 22.1 min), nodakenin (tR 23.4 min), icariin (tR 31.1 min), glycyrrhizin (tR 38.4 min), decursin (tR 49.7 min), and decursinol angelate (tR 50.1 min) were identified by HPLC PDA analysis and comparison with standard compounds. Liquiritin (tR 22.1 min), icariin (tR 31.1 min), and glycyrrhizin (tR 38.4 min) were lower in KIOM-MA128 (fermented KIOM-MA) following deglycosylation during fermentation. Two peaks at retention times of 20.2 and 20.6 minutes increased and one peak at 66.5 minutes appeared. The structures of the two increased constituents and the new constituent were identified by chromatographic separation and spectroscopic techniques (Figures 1 and 2).

### 3.2. KIOM-MA and KIOM-MA128 Exhibited Potent Antiatopic Effect in BALB/c Mice Atopic Dermatitis Model

**Figure 3:**
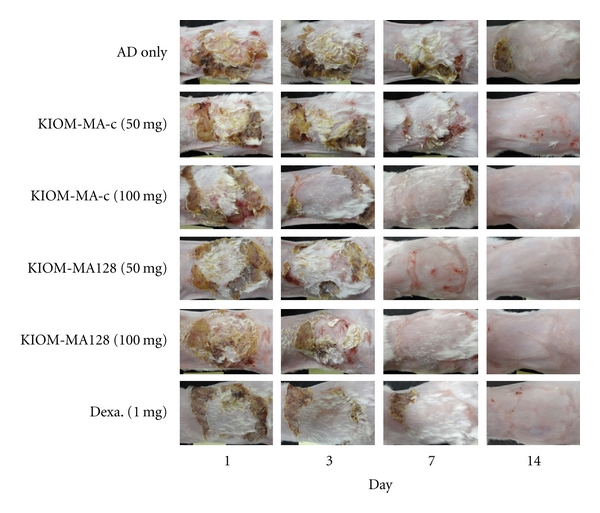
The effect of KIOM-MA and KIOM-MA128 on atopic dermatitis-induced BALB/c mice. KIOM-MA and KIOM-MA128 significantly mitigated five symptoms: erythema/darkening, edema/population, excoriations, lichenification/prurigo, and dryness at 7 days after treatment.

To investigate the effect of KIOM-MA and KIOM-MA128 on AD, 50, or 100 mg KIOM-MA, KIOM-MA128, 1 mg dexamethasone or a control (without KIOM additive) was orally administered to five-week-old BALB/c mice, which have AD induced by ovalbumin and aluminum hydroxide, once a day for consecutive days. The atopic lesions of mice were observed until 14 days after the first treatment. From 7 days posttreatment, both KIOM-MA and KIOM-MA128 remarkably improved atopic symptoms comparable to control dexamethasone, which is well-known anti-inflammatory reagent (Figure 3). Improved five symptoms contain erythema/darkening, edema/papulation, excoriations, lichenification/prurigo, and dryness.

### 3.3. The Effect of KIOM-MA and KIOM-MA128 on Clinical Skin Severity Score

**Figure 4:**
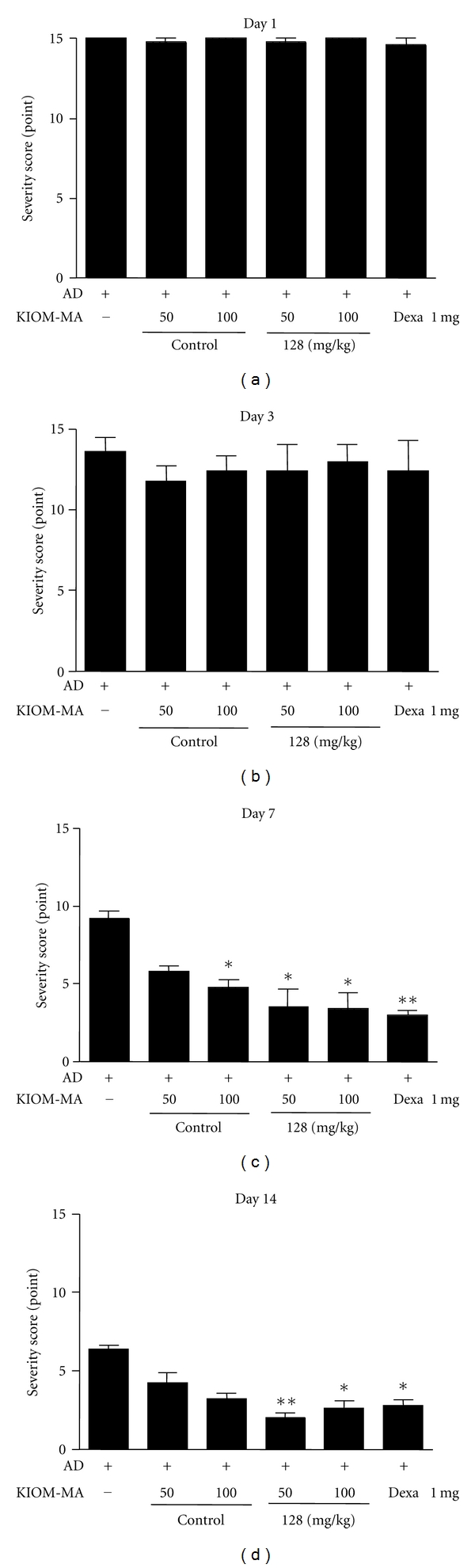
The effect of KIOM-MA and KIOM-MA128 on skin lesions. The improvement in skin lesions was evaluated based on the skin severity score. The clinical skin severity was decreased in the KIOM-MA-treated group and much more improved in KIOM-MA128 after 7 days. Experimental values are given as the means ± SEM (*n* = 5). *P < 0.05, **P < 0.01, when compared with untreated group.

Next, the improvement observed in atopic skin lesions by KIOM-MA and KIOM-MA128 was evaluated based on the skin severity score. The clinical skin severity score was calculated from five major symptoms consisting of symptomerythema/darkening, edema/papulation, excoriations, lichenification/prurigo, and dryness. As shown in Figure 4, skin severity score was significantly decreased in the KIOM-MA-treated group (*P* value = 0.0422 and 0.0487 at 50 and 10 mg/kg, resp., compared with no-treated group) and much more reduced in KIOM-MA128 (*P* value = 0.0282 and 0.0120 at 50 and 10 mg/kg, resp., compared with no-treated group) after 7 days. KIOM-MA and KIOM-MA128 tended to decrease the skin severity score on day 5, and the scores of KIOM-MA and KIOM-MA128 were 5 and 3, respectively, compared with that of negative control that was 10 on day 7 (Figure 4). The skin severity score of dexamethasone was 3, same extent with KIOM-MA128. On day 14, the scores of all groups were decreased, and on day 21, KIOM-MA or dexamethasone-treated group had the same clinical skin score as the no-treatment and the control group (data not shown). Whether to determine if the effect of KIOM-MA or KIOM-MA128 depended on the treatment dose, we administered different doses (50 and 100 mg/kg) of KIOM-MA. We found that both KIOM-MA and KIOM-MA128 reduce the clinical skin severity score in a dose-dependent manner on day 7 (Figure 4).

### 3.4. The Effect of KIOM-MA and KIOM-MA128 on Scratching Behavior

**Figure 5:**
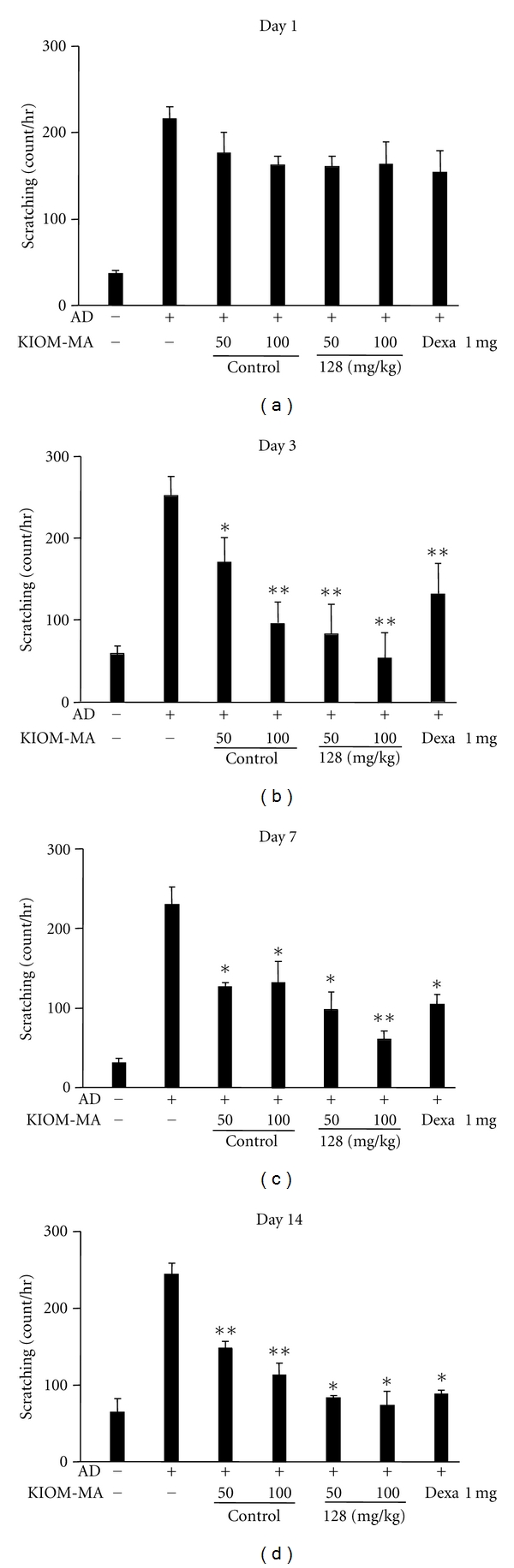
The effect of KIOM-MA and KIOM-MA128 on scratching behavior. KIOM-MA or KIOM-MA128 was administered to the mice as described in the Materials and Methods. Simultaneously, we counted the number of times the mice scratched skin lesions for 30 min on 14 consecutive days. KIOM-MA and KIOM-MA128 caused a decrease in the scratching behavior, which was accompanied by improvement of skin eruptions. *P < 0.05, **P < 0.01, when compared with untreated group.

We next investigated the effects of oral treatment of KIOM-MA on the scratching behavior in the mice. As represented in Figure 5, KIOM-MA reduced the number of scratches and KIOM-MA128 exhibited more significantly decreased scratches on day 3 after the start of treatment, which was accompanied by an improvement in the eruptions (data not shown). On day 7 after treatment, the numbers of scratching in atopic mice by treatment of KIOM-MA or KIOM-MA128 were significantly lower than control dexamethasone. The diminished scratching behavior by treatment of KIOM-MA (*P* value = 0.0197 and 0.02368 at 50 and 10 mg/kg, resp., compared with no-treated group) or KIOM-MA128 (*P* value = 0.0028 and 0.0013 at 50 and 10 mg/kg, resp., compared with no-treated group) was observed in dose-dependent manner. An increase in the scratching behavior of the mice was again observed immediately after the discontinuation of the KIOM-MA treatment (data not shown).

### 3.5. The Effect of KIOM-MA and KIOM-MA128 on IgE Secretion

**Figure 6:**
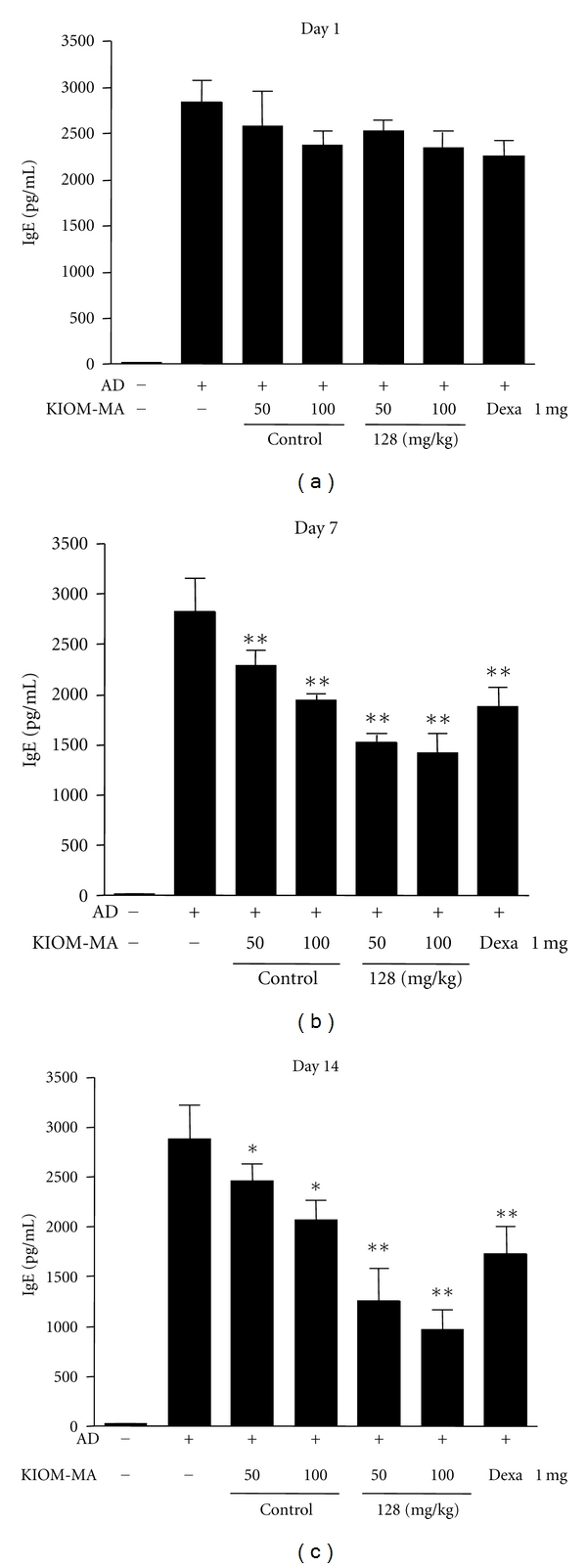
The effect of KIOM-MA and KIOM-MA128 on IgE secretion. A drastic decrease in the IgE levels was observed in KIOM-MA and KIOM-MA128-treated mice. Values are the mean ± SEM (*n* = 5) *P < 0.05, **P < 0.01, when compared with the no-treatment group.

Because AD is a type I IgE-mediated hypersensitivity reaction contributing to immune dysregulation and its major characteristics is hyperproduction of IgE, we examined the plasma IgE levels in the mice treated with KIOM-MA or KIOM-MA128 and compared with that of control.  Figure 6 shows that when the mice were induced to the AD, the plasma IgE concentration was highly increased than control group, and the IgE levels increased in the AD mice were reduced with KIOM-MA (*P* value = 0.0090 and 0.0051 at 50 and 10 mg/kg, resp., compared with no-treated group) or KIOM-MA128 (*P* value = 0.0019 and 0.0023 at 50 and 10 mg/kg, resp., compared with no-treated group) treatment from day 7 after treatment. Consistent with skin severity and starching behavior results, KIOM-MA128 exhibited much stronger reducing effect on IgE secretion in the plasma of AD mice model (Figure 6).

## 4. Discussion

We investigated the effects of KIOM-MA and its fermented product, KIOM-MA128,* in vitro* and* in vivo* in an AD-like animal model. The results demonstrated that KIOM-MA effectively reduced the clinical features and the index of AD-like serum IgE level. Considering that AD is the most common skin disease, it is worth noting that the clinical recovery from AD-like skin was more clearly seen based on macrography, scratching count, and severity scores in the treatment group. Hyperpigmentations and erythematous lesion eruptions also gradually improved significantly in the treatment group in a dose-dependent manner. The mice treated with KIOM-MA or KIOM-MA128 did not show any clinical signs in the skin after seven days of oral administration comparable to dexamethasone-treated group (positive control group). The no-treatment group suffered continuously from skin eruptions and skin injuries in response to scratching behavior after seven days. Furthermore, KIOM-MA and KIOM-MA128 significantly inhibited the AD clinical signs in a dose-dependent manner. However, topical application routes for atopic therapy are needed and are being tested now. The pharmacodynamics and action mechanisms of KIOM-MA and KIOM-MA128 are currently being investigated *in vitro* with a focus on Th1/Th2 cytokines and related chemokines.

Several previous studies have evaluated the use of glycyrrhizin [20], liquiritin [21], and decursin [21] for the treatment of AD, as well as nodakenin for anti-inflammatory effects [22], icariin for immunoregulatory effects [23], and decursinol angelate for blocking inflammatory activation [24]. Our results indicate that the high concentration of several constituents including glycyrrhizin contained in the KIOM-MA may be responsible for anti-inflammatory effect of KIOM-MA and KIOM-MA128. Interestingly, the KIOM-MA128 fermented from KIOM-MA by *L. acidophilus* more significantly improved atopic symptoms. Fermentation, a progress of decomposition of organic matter occurred by *L. acidophilus,* generates various low molecular weight substances like aglycone from macromolecule including glycoside. This fact implies that active aglycone formed during fermentation is more infiltrative than glycoside *in vivo*. Aglycones may increase the skin or mucosal permeability, thus, optimizing the physiological bioavailability *in vivo*. In addition, HPLC-DAD analysis of KIOM-MA and KIOM-MA128 strongly implies that new constituents or increased constituents may be involved in the antiallergic mechanism. However, further studies should be performed to test these hypotheses. Two increased peaks and one new peak are now being subjected to isolate for the further study.

In summary, the pharmacological profiles of KIOM-MA and KIOM-MA128 were as follows: the reduction of clinical features and IgE production suggest that KIOM-MA and KIOM-MA128 are effective for the treatment of AD in a dose-dependent manner. The oral administration route may be useful for clinical improvement of AD, even though topical application is generally thought to have more therapeutic advantages. Bioconversion by fermentation significantly enhances the anti-inflammatory effect of KIOM-MA. Further studies are necessary to clarify the mechanisms of KIOM-MA and KIOM-MA128 for the treatment of AD. Nevertheless, our findings suggest that KIOM-MA and KIOM-MA128, which has stronger efficacy, could be applicable to develop potent therapeutic reagent for the treatment of AD.

## Figures and Tables

**Table 1:** Mobile phase condition of chromatographic separation (254 nm).

Time (min)	Water (in 2% acetic acid)	Acetonitrile (in 2% acetic acid)	Flow rate (mL/min)
0	95	5	1
5	95	5	1
60	20	80	1
70	20	80	1
75	95	5	1
90	95	5	1
