# Erratum to “Effectiveness of the Novel Herbal Medicine, KIOM-MA, and Its Bioconversion Product, KIOM-MA128, on the Treatment of Atopic Dermatitis”

**DOI:** 10.1155/2014/402092

**Published:** 2014-02-05

**Authors:** Tae Ho Chung, Tae Jin Kang, Won-Kyung Cho, Ga Yeong Im, Geum Seon Lee, Min Cheol Yang, Chang-Won Cho, Jin Yeul Ma

**Affiliations:** ^1^Center for Herbal Medicine Improvement Research, Korea Institute of Oriental Medicine (KIOM), 483 Expo-ro, Yuseong-gu, Daejeon 305-811, Republic of Korea; ^2^College of Pharmacy, Sahmyook University, 26-21 Kongnung 2-dong, Nowon-gu, Seoul 139-742, Republic of Korea; ^3^Regional Food Industry Research Group, Korea Food Research Institute, Sungnam 463-746, Republic of Korea

In the whole paper, *Lactobacillus acidophilus* was incorrectly used. The correct name is *Lactobacillus rhamnosus*. The authors apologize for this error.

